# Retraction: AptaFluorescence: An aptamer-based fluorescent imaging protocol for biomolecule visualization

**DOI:** 10.1371/journal.pone.0356046

**Published:** 2026-08-14

**Authors:** The PLOS One Editors

Following the publication of this article [1], concerns were raised about Figs 2-4. Specifically:

1. The following panels appear to partially overlap:
  - Fig 3B: the left and middle +Dox panels.
  - Fig 4B: the middle +Dox panel and right -Dox panel.
2. There appear to be inconsistencies in the fluorescence signals presented in Fig 2:
  - The intensity of the fluorescence signal in the 63X IgG antibody panels appears to be inconsistent with the intensity of the fluorescent signal observed in the corresponding original raw image files underlying these results [2].
  - The reported increase in signal for the + Dox condition compared to -Dox appears inconsistent across magnifications.
  - The signal intensity in the c-Myc aptamer images appears similar to the signal intensity of the non-targeting NT aptamer containing a random sequence.
3. In Figs 2-4, fluorescence intensity is compared, but the exposure times used for image acquisition are not reported, which precludes assessment of whether differences in signal reflect true biological variation or differences in imaging conditions.
4. Concerns have been raised about the reliability of the quantification of the fluorescence results presented in Figs 3 and 4:
  - The study used technical replicates rather than biological replicates to quantify differences in fluorescence intensity between the -Dox and +Dox conditions.
  - Only three images were used for the quantitative comparison with the number of cells per image varying from 2 to 25.
5. The article appears to infer specificity of the aptamer from the higher signal observed in the +Dox condition compared with the -Dox condition. However, given that c-Myc is a transcription factor, the data presented do not exclude the possibility that its overexpression in this system may result in altered expression of other proteins, and therefore do not unambiguously establish that the observed signal is specific to c-Myc.

The first author stated that an error was made in the preparation of Figs 3 and 4 which impacted the quantification and subsequent statistical analysis. They provided an updated version of Fig 3 and Table 1 and stated the overall conclusions of the article were not affected.

They noted that for Fig 2, brightness and contrast adjustments had been applied uniformly across each entire image and the relevant controls. They also stated that exposure time was adjusted independently for each magnification and staining condition to obtain representative images, and that exposure settings therefore differ between panels and are not intended for direct comparison across staining conditions.

They further noted that within each magnification and condition, the same exposure settings were maintained for +Dox and -Dox, allowing comparison between treatment conditions under identical acquisition settings.

The first author provided replacement images from repeat experiments. Editorial assessment of these data raised additional concerns since the c-MYC aptamer image data appeared to have been acquired using a longer exposure time than the NT aptamer images, further calling into question the article’s findings regarding the specificity of the c-MYC aptamer.

The concerns described above, including issues related to image integrity, inconsistencies in the reported signal, the specificity of the aptamer, and the quantitative analyses, were assessed by an independent expert who confirmed the validity of the identified issues and concluded the author explanations and replacement images did not fully resolve the concerns. In light of the unresolved concerns, which call into question the reliability and validity of the results and conclusions presented in [1], the *PLOS One* Editors retract this article.

MJF agreed with the retraction. CCYL did not agree with the retraction.
